# Recent Advances in the Use of Stem Cells in Tissue Engineering and Adjunct Therapies for Tendon Reconstruction and Future Perspectives

**DOI:** 10.3390/ijms25084498

**Published:** 2024-04-19

**Authors:** Paweł Dec, Małgorzata Żyłka, Piotr Burszewski, Andrzej Modrzejewski, Andrzej Pawlik

**Affiliations:** 1 Plastic and Reconstructive Surgery Department, 109 Military Hospital, 71-422 Szczecin, Poland; pawel_dec@onet.pl (P.D.); mzylka96@gmail.com (M.Ż.); piotrburszewski@gmail.com (P.B.); 2 Department of Surgery, Pomeranian Medical University, 71-422 Szczecin, Poland; amodrzejewski@interia.pl; 3 Department of Physiology, Pomeranian Medical University, 70-111 Szczecin, Poland

**Keywords:** tendon, stem cells, tissue engineering, reconstruction

## Abstract

Due to their function, tendons are exposed to acute injuries. This type of damage to the musculoskeletal system represents a challenge for clinicians when natural regeneration and treatment methods do not produce the expected results. Currently, treatment is long and associated with long-term complications. In this review, we discuss the use of stem cells in the treatment of tendons, including how to induce appropriate cell differentiation based on gene therapy, growth factors, tissue engineering, proteins involved in regenerative process, drugs and three-dimensional (3D) structures. A multidirectional approach as well as the incorporation of novel components of the therapy will improve the techniques used and benefit patients with tendon injuries in the future.

## 1. Introduction

A tendon is an anatomical structure that connects muscle to bone. It is made up of collagen fibres of various sizes that fit tightly together. Tenocytes produce an extracellular matrix (ECM) mostly composed of collagen and proteoglycans. This structure ensures the strength of these structures against mechanical forces. Unfortunately, due to their function and the need to endure high mechanical loads, tendons are prone to injury. Indeed, tendon injuries are common and represent a financial challenge throughout the world. It is estimated that more than 30 million tendon-related procedures are performed, costing more than EUR 150 billion in United States and Europe [1,2,3]. In this review, we focus on molecular handles, the use of stem cells in the process of tendon regeneration and the latest discoveries in this field.

Physiological tendon regeneration is inefficient and prolonged. The symptoms of acute tendon injury include the onset of acute pain, reduced muscle strength and restricted mobility. Due to an inadequate blood supply and low cellular metabolism, a tendon has a limited regenerative capacity [4,5,6]. Injury stimulates the formation of adhesions between the tendon and the surrounding tissues, which can lead to the formation of asymmetrical scar tissue that interferes with tendon function and increases the risk of re-injury [7,8,9]. The tendon healing process is divided into three stages: inflammation, proliferation and remodelling [10], which occur in succession. During the first stage, inflammatory cells and exogenous fibroblasts at the site of injury secrete large amounts of cytokines that stimulate cell migration and neovascularisation. Tenocytes and macrophages play a major role in the second stage, proliferation. This involves fibroblast proliferation, increased synthesis of ECM composed mainly of type I collagen [11], activation of tendon stem/progenitor cells (TSPCs) and tendon formation. Stem cells are characterised by their ability to differentiate into various cell lineages and are involved in tendon repair by regulating inflammation and forming new tissue at the site of injury, among other actions. In vitro and in vivo animal studies have shown that TSPCs differentiate into tenocytes (tendon cells) to support the tendon repair process [12]. Tendon adhesion is negatively affected by an imbalance between endogenous and exogenous tendon regeneration pathways, which are regulated by tenocytes and fibroblasts [13,14]. Endogenous pathways oversee tenocyte differentiation and proliferation and inhibit adhesion formation in a tendon. Exogenous pathways, on the other hand, stimulate fibroblast proliferation, contributing to the formation of scar tissue and adhesions, negatively affecting the mobility of tendon tissue. The dominance of exogenous pathways stimulates the formation of tendon adhesions, but a lack of integrity, local inflammatory response and impaired blood supply are among the causes [9,10,11].

Unfortunately, the currently available treatments, both conservative and surgical, are unable to restore the original structure, functionality and biomechanical properties of a tendon. Therefore, new therapies are needed to aid the regeneration process so that the newly formed tissue has physiological properties that are as close as possible to those of a tendon. Therefore, it is necessary to search for more effective methods that utilise stem cells, gene therapy, growth factors, tissue engineering [12], proteins involved in the regenerative process and/or three-dimensional (3D) structures (produced with methods such as 3D printing) that induce appropriate cell differentiation. In this review, we discuss the available evidence regarding promising methods and molecular handles that may have a positive impact on tendon healing after acute mechanical injuries.

## 2. Stem Cells and Their Properties

**Figure 1:**
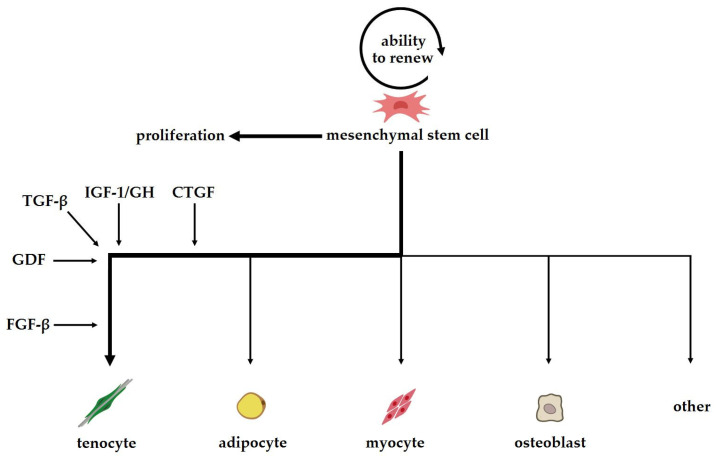
Scheme of the differentiation of mesenchymal stem cells.

Stem cells differ from other cells based on their ability to renew, expand and differentiate (Figure 1) into multiple cell lines [15]. They also have high biological activity and the capacity for unlimited proliferation. Moreover, through paracrine stimulation, they can exert immunomodulatory effects to stimulate tendon healing. For this reason, the use of stem cells for repair is a promising area of research [16].

### 2.1. Types and Sources of Stem Cells

Various types of mesenchymal stem cells (MSCs), including TSPCs, are obtained from various tissues, including tendons, adipose tissue, blood and umbilical cord tissue [17,18,19,20,21,22]. In a study involving rats with artificially created full-thickness damage to the supraspinatus muscle tendon, researchers showed that the place from which stem cells are obtained has a significant impact on their ability regarding and participation in the regeneration process. The authors assessed the potential of MSCs from three different sources: umbilical cord blood, umbilical cord tissue and bone marrow. Under the same 3D conditions, the cells achieved different levels of similarity to the structure of a healthy tendon, with umbilical tissue cells showing better expression of tendon-related genes and ECM production [23]. In the following subsections, we discuss different types of stem cells in terms of their potential to regenerate tendon tissue.

#### 2.1.1. Tendon Progenitor Stem Cells (TSPCs)

TSPCs exhibit the typical characteristics of MSCs. They have the ability to regenerate and differentiate into a variety of cell lines (adipogenic, osteogenic and chondrogenic) and can stimulate clonogenesis. Moreover, TPSCs express many tendon-related genes, which are related to their tendency to differentiate into tissue that is similar in structure to tendons and cells found in tendon attachments (enthesis-like tissue) [24]. In a study involving horses [25], autologous TSPCs stimulated the regeneration of flexor tendons with higher tensile strength. These findings indicate that TSPCs can be used to improve the regeneration process. However, TSPCs are not without drawbacks that make it difficult to use them on a large scale to treat tendon injuries. The small number of these cells in tendon tissue means that a large amount of source material is necessary to obtain a sufficient number of TSPCs. A study using TSPCs from rat tail tendons showed that difficulties in obtaining these cells may translate into their low ability to proliferate [26]. Moreover, the donor site is susceptible to secondary injuries, a factor that limits the clinical use of TSPCs. An additional negative phenomenon is phenotypic drift [27], which reduces the regenerative potential of the tendon tissue that uses these cells. Another study showed that negative phenotypic drift can occur with the use of TSPCs [26].

#### 2.1.2. Adipose Tissue-Derived Stem Cells (ASCs)

ASCs morphologically and molecularly resemble bone marrow-derived mesenchymal stem cells (BMSCs). A major advantage of ASCs is the ease of obtaining them from the donor body via liposuction of subcutaneous adipose tissue. After connective tissue growth factor (CTGF) stimulation, animal ASCs have increased expression of scleraxis (Scx) and tenomodulin (Tnmd) [28], and the new neo-tendon has a histological structure similar to a tendon and physiological tensile strength [29]. In human studies, ASCs treated with bone morphogenetic protein 12 (BMP12) differentiated into tendon cells with increased Scx expression [30]. In contrast, another study conducted histological and biomechanical analyses on supraspinatus rotator cuff tendons [31]. The authors tested three treatments: surgical supply, collagen stimulation and collagen stimulation with ASCs. Histological examination revealed a reduction in oedema, a lower neutrophil count and less inflammation in the group treated with ASCs. There were no differences in the biomechanical results in the other groups. A disadvantage of ASCs is their tendency to undergo adipogenesis. Indeed, animal experiments have shown fat deposition during the tendon regeneration process [32]. Additional research is necessary to understand the potential drawbacks of ASCs.

#### 2.1.3. Bone Marrow-Derived Mesenchymal Stem (BMSCs)

Many studies have shown that BMSCs also have great potential to differentiate into tendons [33,34,35]. Scx and Tnmd expression in BMSCs, stimulated by growth factors such as BMP14, induces their differentiation into tendon cells in vitro [33]. They are easy to obtain [34], although compared with ASCs, a larger volume of source is needed to obtain the same number of BMSCs [36]. Nevertheless, they are still technically easier to obtain than TSPCs. The results of a 12-month study involving participants with patellar tendinopathy showed that BMSC biologic treatment was uncomplicated and safe. The participants showed significant clinical improvements and the tendons examined by magnetic resonance imaging (MRI) showed a more organised structure [37]. Unfortunately, the disadvantage of BMSCs is an increased incidence of ectopic bone formation in the animal’s tendons and reduced strength compared with tendons that were not exposed to injury [38]. Additionally, the aspiration process is painful, which can cause additional unpleasantness for patients [39].

#### 2.1.4. Embryonic Stem Cells (ESCs) and Pluripotent Stem Cells

ESCs are obtained from embryos. Their uniqueness lies in their ability to differentiate into all cell lineages from all embryonic cotyledons: mesoderm, ectoderm and endoderm. In vitro studies have shown that human ESCs tend to transform into tendon cells under the influence of exogenous BMP12 and BMP13 in the presence of ascorbic acid [40]. Gene transcription analysis of newly formed cells showed the expression of the TNC, DCN, TNMD, THBS4, COL1A2 and COL3A1 genes, which are known to be associated with tenocytes. The effect was not visible when dorsomorphin, a compound that blocks the Smad cascade, was added to the mixture. DCN and TNC gene expression and tendon matrix formation were inhibited. The targeted in vitro production of tenocytes from pluripotent stem cells creates optimistic expectations for the use of similar methods in future techniques aimed at more efficient stimulation of tendon tissue regeneration [40]. However, the use of ESCs raises moral questions because of the way they are obtained from embryos. Another very significant problem is that there is an increased risk of carcinogenesis with the use of these cells [41].

Induced pluripotent stem cells (iPSCs) present a solution to the moral problems associated with the use of ESCs, as the source of derivation of this type of cells is differentiated somatic cells [42,43], addressing ethical concerns. The potential of these cells was demonstrated in a study in which repetitive uniaxial mechanical loading of iPSCs led to increased Scx expression [42], confirming that they could be used to stimulate tendon regeneration. Unfortunately, complicated and inefficient protocols for obtaining these cells and the still real risk of neoplastic transformation [44] cast doubt on iPSCs as the best cells for use in future tendon injury treatment strategies (Table 1). 

## 3. The Role of Growth Factors, Transcription Factors and Other Biological Mediators in Stem Cell Differentiation and Tendon Regeneration

### 3.1. Growth Factors and Bioactive Proteins in Tendon Regeneration

Many growth factors and proteins are involved in tendon regeneration and the differentiation of stem cells into tendon cells. Their influence on this complex process is still being investigated. In rats, polyglycolic acid (PGA) and type I collagen scaffolds were used and attached with a frame suture to ensure postoperative functional continuity. In the study group, the scaffolds were seeded with MSCs or tenocytes; the control group did not have cells. The samples using tenocytes had significantly better strength compared with MSCs and the control group, while the maximum strength to resist failure was similar in all groups [45]. These findings suggest that the presence of differentiated tenocytes has a direct impact on the quality of the regenerating tendon. Because the process is complex, there are many factors that trigger the differentiation of stem cells into other cell lines [45,46]. In the following subsections, we describe a few of them.

### 3.2. Transforming Growth Factor Beta (TGF-β)

TGF-β activates the most important signalling pathways during tendon cell differentiation and formation [47,48,49,50]. It plays a key role in promoting MSC tenogenesis [48] and is also involved in inflammatory responses, angiogenesis, collagen synthesis and fibrosis or excessive scarring during tendon healing [51,52,53]. All TGF-β isoforms have been shown to play a role in tendon development and differentiation [54]. TGF-β2 and TGF-β3 are considered essential factors for tendon development and can induce differentiation of tendon stem cells [55,56]. These TGF-β ligands mainly transmit biological responses through the Smad2/3 intracellular pathway [47,52,57,58]. In terms of gene expression, application of TGF-β3 to tendon cells can increase Smad7 levels and decrease Smad3 levels, thereby reducing scarring and tendon adhesion, which promotes tendon repair [59]. Similarly, Smad3 suppression may play a role in rotator cuff repair [60]. TGF-β1 signalling is required to maintain Scx expression in cultured tenocytes. TGF-β2 influences the acquisition of fibroblastic morphology by stem cells and increases the expression of Scx and Tnmd [49]. TGF-β is also essential during the tendon repair process: based on in vivo experiments, it produces tissue with better biomechanical parameters and histological structure [61].

Although TGF-β plays a key role in tendon repair, it can also, through activation of the TGF-β1/Smad signalling pathway, lead to progressive fibrosis and scar formation [62,63] and can even induce apoptosis [64]. TGF-β1-stimulated scarring is associated with increased deposition of fibronectin and type I and III collagen, confirming that tendon repair through scar tissue underlies tendon adhesion formation [65,66,67]. Two major target regulators, Smad2 and Smad3, promote TGF-β1-mediated tissue fibrosis. Studies have shown that TGF-β1 inhibition can reduce scarring while decreasing the biomechanical strength of repaired tendon sites. Moreover, administration of an antibody that inhibits TGF-β1 in rabbit flexor tendons reduced the incidence of adhesion phenomenon along with improving the morphology of the tendon structure. Inhibition of this antibody reversed the effect [68].

Studies using exogenous TGF-β3 have also provided interesting results [69]. External delivery of TGF-β3 improved tendon structure and mechanical properties after Achilles tendon injury in rats [70]. BMSCs transfected with TGF-β1 complementary DNA (cDNA) significantly improved the biomechanical properties of the injured rabbit Achilles tendon [71].

Because of the number of pathways activated by TGF-β and the conflicting roles it plays, the direct effect of this growth factor in tendon regeneration is not clear. Numerous studies have attempted to resolve this ambiguity. One study compared differences in tendon healing with or without TGF-β stimulation. The authors showed that Scx-negative tenocyte proliferation and recruitment of subsequent tenoblasts and functional regeneration depended on TGF-β signalling, while early Scx-positive tenocyte proliferation did not [72]. Due to the multitude of pathways activated by TGF-β and the conflicting roles it plays in tendon tissue regeneration, the direct effects of this growth factor signalling on tendon regeneration are not clear and additional detailed research is required. Nonetheless, TGF-β is clearly one of the most promising growth factors.

### 3.3. Growth Differentiation Factors (GDFs)

GDFs is a part of the TGF-β group and is involved in the development of the musculoskeletal structures, including tendons [73,74]. In one study, researchers severed the Achilles tendon of rats in order to examine influence of GDFs and loading on the tendon regeneration process. During tendon healing, the expression of GDF5, GDF6 and GDF7 was observed, confirming that these growth factors play a role in tendon regeneration [75]. Other research, however, showed that the expression of GDFs decreases during tendon regeneration [76]. Immunoassays revealed high expression of all controlled growth factors at 1 week and a decrease to undetectable levels by 16 weeks. Thus, it can be concluded that their role is crucial during the early stages of tendon healing. In a subsequent study, GDF8 led to increased expression of tendon markers and tendon differentiation of mouse myocyte cell (2C12) [77]. Immunohistochemistry showed that GDF8 inhibited myotube formation and promoted the formation of spindle-shaped cells expressing tenomodulin. Silencing of the Smad3 pathway by usage of RNAi suppressed tenomodulin expression. These results suggest that GDF8 plays a role in the induction of tenogenic differentiation of C2C12 through the Smad3-mediated pathway. In another study, rat BMSCs were stimulated with GDF8, which led to Scx, type I collagen and Tnmd expression [78]. This suggests that GDF-8 can influence BMSCs’ growth and differentiation toward a tenocyte lineage. Moreover, in a study on human MSCs, GDF5 could promote tendon differentiation and exhibited a better balance of pro-inflammatory and anti-inflammatory cytokines [79]. The anti-fibrotic properties of GDF5 have also been demonstrated. Transplanted flexor tendons treated with different GDF5 doses showed comparable improvements in joint flexion function, with lower GDF5 doses more efficiently inhibiting adhesion and fibrosis [80]. The abovementioned studies prove that GDFs have a significant impact on the tendon regeneration process.

### 3.4. Connective Tissue Growth Factor CTGF

CTGF is known to be able to promote fibroblast proliferation. Moreover, studies revealed that CTGF is able to induce BMSCs to differentiate into fibroblasts [81]. BMSCs lose their surface mesenchymal epitopes and produce collagen type I and tenacin-C as a result of CTGF stimulation. CTGF also exerts its effect through activation of the focal adhesion kinase (FAK)/extracellular signal-regulated kinase 1/2 (ERK1/2) pathway in TSPCs, which positively affects tendon regeneration [82]. Treatment of mouse ASCs with CTGF promoted the proliferation of damaged tendon cells and induced the expression of tendon markers such as Scx and Tnmd [28]. The tendon repair potential of CTGF has also been confirmed in in vivo studies. The CD146 receptor is a specific marker of TSPCs [24]. In vivo administration of CTGF during the early phase of tendon repair induced proliferation and differentiation of CD146-positive cells into tenocytes [82]. CTGF promoted the differentiation of TPSCs with significantly increased expression of collagen I, Tnmd, Scx and Tnc. Moreover, these effects improved tendon repair and led to better structural reconstruction of the tendon and restoration of function. A subsequent search investigated if CTGF was required for tendon differentiation of rat TSCs stimulated by BMP12. TSCs presented tenogenic differentiation markers under the influence of BMP12 and CTGF promoted this effect via the Smad1/5/8 pathway. Tendon differentiation was not present in the occurrence of knockdown of CTGF expression. These results suggest that CTGF plays an important role in this process and may be further investigated for treatment purposes [83]. The results of study that is mentioned in genetic modification section also investigated influence of BMP12 and CTGF on TDSCs. It showed that BMP12 and CTGF transfection leads to tenogenic differentiation of TDSCs by upregulating tenogenic gene expression [84]. Simultaneously, downregulation of osteogenic, adipogenic and chondrogenic gene expression was present. The influence of BMP12 and CTGF stimulated rat patellar tendon window defect regeneration.

### 3.5. Fibroblast Growth Factor (FGFs)

FGFs play an important role differentiation and proliferation processes of cells [84]. FGF2 is a potent mitogen [85,86,87]. Through activation of the FGF/ERK/mitogen-activated protein kinase (MAPK) pathway, it regulates the differentiation of MSCs and progenitor stem cells towards tendon tissue [88]. Lower FGF2 concentrations, below 10 ng/mL, were more beneficial for tendon differentiation than higher concentrations [89], a phenomenon similar to GDFs. Human TSPCs infected with a viral vector containing FGF2 induced hyperexpression of this factor and led to increased type III collagen and Scx levels, which are essential for tendon regeneration [90]. Another study performed on rats found that increased expression levels of tendon-related markers such as Scx and Tnmd were detected in the FGF-2-treated group at between 4 and 12 weeks. This proves that FGF-2 molecules stimulate growth of TSPCs and improve biomechanical and histological repairs of newly formed tissue [91]. There were less promising results from a study involving FGF8b stimulation: it enhanced the tendency towards chondrogenic differentiation and reduced the expression of tendon ECM (type I and III collagen) and Scx, Tnmd and Tnc. These results confirm the multifaceted nature of the processes that occur during regeneration. The effects of FGFs on tendon differentiation require further investigation.

### 3.6. Insulin-Like Growth Factor (IGF-1) 

IGF-1 is a hormone produced in the liver in response to growth hormone (GH) stimulation [92,93]. IGF-1 has an insulin-like structure and binds to the IGF1 receptor as well as the insulin receptor; insulin can also bind to both receptors [94]. This protein hormone is responsible, among other things, for cell differentiation, proliferation and protein synthesis. There are three isoforms of IGF-1: Ea, Eb and E [95]. IGF-1Ec is highly force-sensitive [96]. It also functions as a crucial regenerative agent, stimulating the activation of stem cells [97,98]. 

The binding of IGF-1 to the IGF1R receptor controls various pathways. Upon IGF-1 binding, receptor tyrosine kinases (RTKs) are activated [99]. The tyrosine residues of RTKs are associated with IGF-1 receptor β subunits and are phosphorylated upon binding of IGF-1 to the IGF-1 receptor [100]. IGF-1 triggers [101,102] several pathways, such as phosphoinositide 3-kinase (PI3K)/Akt [103], Ras-MAPK [104] and phospholipase C (PLC) [105] pathways. In adult tendons, IGF-1 modulates tenocyte proliferation and collagen synthesis through the PI3K/Akt/mTOR pathway [106] and indirectly via TGF-β1 [107].

Research indicates that IGF-1 demonstrates anabolic effects by promoting the synthesis of DNA and collagen. Its significance lies in stimulation of tendon growth in adults in response to stress [106]. In vitro and in vivo studies have been concluded to examine the influence of IGF-1 on tendon tissue [108,109]. In vivo, growth factors have been exposed to various substances in the human body such as enzymes and fluids that can have influence on the mechanism of action of protein. In addition, sterilisation of the material can lead to degradation [110] and a reduction in the bioactivity of the incorporated growth factors [111,112]. The overall effect of IGF-1 on cellular metabolism can be summarised as enhanced anabolism and reduce catabolism. In addition, it synergistically increases the effect of GH on lipolysis and ketogenesis [113,114]. IGF-1 is also involved in inhibiting ECM degradation. IGF-1 has been documented to decrease the release of glycosaminoglycans (GAGs) from the ECM [115] and to prevent the release of collagen from the ECM [116]. Another study reported that IGF-1 administration inhibited the loss of sulphated GAGs and collagen [117]. Furthermore, IGF-1 was found to inhibit cell apoptosis induced by collagen release from the ECM [118]. In vivo research has demonstrated that IGF-1 accelerates the healing process, increases cellular DNA content and enhances the biomechanical properties of tendons [119,120,121]. Conversely, histopathological examinations did not reveal significant structural disparities between tissues stimulated with IGF-1 and a control group.

Researchers have used several methods to deliver IGF-1 to tissues. One common way is to inject IGF-1 into the injury site [122] or to inject GH, which stimulates IGF-1 release [121,123]. In one study, the rat tibial tendon was excised and the Achilles tendon was cut transversely [109]. The authors showed that the time to recovery of function was shortened with an additional injection of IGF-1. A study in a horse model of flexor tendonitis [119] showed that there was less swelling in the IGF-1-treated group and damage, as assessed by ultrasound, was less, especially after 4 weeks. Mechanical tests showed a tendency towards tendon sclerosis in the IGF-1 group. A significant discovery from this research was the increased accumulation of DNA and hydroxyproline, indicating a higher collagen content in the treated group. In a study on human tendons, the positive effect resulting from IGF-1 injection was not confirmed [124]. However, in studies with IGF-1 administration, it was shown that after a short period from the last injection (1–2 h), IGF-1 concentrations in the intercellular matrix were increased in the lower limbs of IGF-1-treated subjects compared with control subjects. The fractional type I collagen synthesis rate in the tendon was significantly higher compared with the control group, but the difference between the two rates varied among patients. Type I procollagen, which is an indirect marker of collagen I synthesis, also increased in the IGF-1 group [122]. 

In addition to the administration of GH and IGF-1 through injection, an established technique is to incorporate GH into fibrin gel, which is then applied to the resulting wound. Researchers assessed the impact of combination IGF-1 and TGF-β1 on the mechanical properties and histology of rabbit knee tendon [120]. It showed this combination was found to enhance the early phase of tendons healing with increased angiogenesis. [120,125]. Angiogenesis can also be stimulated by factors produced by wound macrophages. Wound macrophages produce various growth factors, such as platelet-derived growth factor (PDGF), vascular endothelial growth factor (VEGF) and IGF-1, to alleviate hypoxia during injury and the early phase of healing [126,127]. IGF-1 has been reported to promote proliferation of T cells, to slow neutrophil apoptosis and to stimulate B cell differentiation [128]. 

Histological analysis in studies using platelet-rich plasma (PRP) on rabbit tendons revealed differences between the control and PRP-treated groups. By week 3, the control group developed more immature tissue, while the tissue from the PRP group was denser, contained fewer elastic fibres and had more organised tenocytes. By week 4, the PRP-treated tendons were completely recovered in contrast to the control group [129]. Another study utilising PRP revealed its influence on the morphology of cell. Due to the addition of PRP, tenocytes displayed mainly elongated shapes and cellularity was higher than in the other groups. However, cells of various shapes and sizes, along with fragmented collagen bundles, were observed [130]. 

Another possibility for increasing the contribution of IGF-1 to tendon repair is the use of an IGF-1-embedded matrix. In a study on IGF-1 pegylation, the researchers used a copolymer matrix based on poly(L-lactide)-copoly(ε-caprolactone) with various modifications of embedded IGF-1 in a rat model of a rotator defect [131]. Application of the PEG-IGF-1m matrix led to enhanced in the biomechanical properties of the tissue. Moreover, histological analysis showed an improvement in healing after 8 weeks. 

In summary, promising stimulatory effects of IGF-1 have been demonstrated in tendon tissue. Its effect on MSCs is relatively modest and can only be improved by combining IGF-1 with other growth factors. IGF-1 can be used to stimulate tendon healing because of its anabolic effects.

### 3.7. Yes-Associated Protein (YAP)

The transcriptional co-activator YAP, an important mediator of stem cell regulation, plays an important role in tendon differentiation [132]. Researchers used murine multipotent progenitor cells (C3H10T1/2) and induced their differentiation towards tendon cells with BMP12. Then, they examined the function of YAP in the regulation of tenogenesis. BMP12 increased the expression of YAP and tendon-related proteins such as tenomodulin and tenascin C. The reverse process, reducing YAP levels, inhibited the observed differentiation. These results indicate that YAP is required for the expression of tenogenic markers during tendon differentiation of murine progenitor cells. Therefore, YAP is a novel regulator of BMP12-induced MSC tenogenesis. Although these results demonstrate a potentially promising role for the YAP in tendon healing and regeneration, further investigation, including research involving human stem cells, is essential.

### 3.8. Wnt Ligands

The Wnt signalling pathway plays a significant role in development and growth of various tissues, including tendon tissue. One research revealed that Wnt/β-catenin signalling mediated expression of tenomodulin, which has been recognised as a biomarker for tendon differentiation [133]. In this study other tenomodulin-regulating transcription factors such as scleraxis and Mohawk were evaluated. It was revealed that these transcription factors were not affected. This proves that the Wnt/β-catenin signalling pathway may be a new regulation pathway for tenomodulin expression. The Wnt proteins activate two signalling pathways contingent on their reliance on transduction through β-catenin: the canonical and the noncanonical Wnt pathway [134]. Wnt5a is a classic ligand for the noncanonical Wnt pathway. Wnt5 and Wnt4 also exert effect on MSCs. They mediate tendon differentiation induced by mechanical stimulation [135]. Future studies on the effects of exogenous Wnt on tendon differentiation and tendon repair are worthwhile.

### 3.9. Genetic Modification

**Figure 2:**
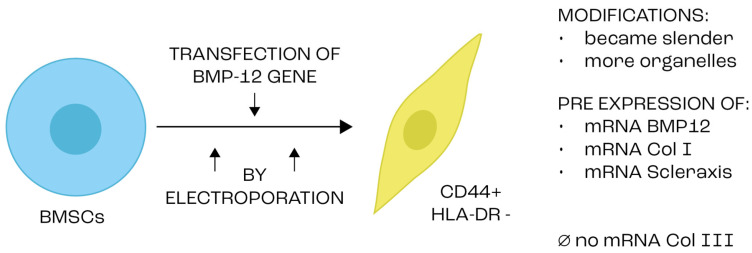
Scheme of the differentiation of tendon stem/progenitor cells under the influence of genetic modification and connective tissue growth factor.

Genetic modification can also be used to stimulate tendon differentiation. Researchers implanted TSPCs into the damaged patellar tendon after infecting it with a recombinant adenovirus containing BMP12 and CTGF [136]. There was increased expression of Tnc, Scx and type I and III collagen. TSPCs with high expression of BMP12 and CTGF stimulated tendon repair in vivo. In another study, researchers transfected MSCs with the BMP12 gene by electroporation. The modified cells showed an altered morphology: they became more slender and formed a network. In addition, they expressed BMP12 and type I collagen messenger RNA (mRNA), but not type III collagen mRNA. The newly formed cells were positive for CD44 but negative for HLA-DR (Figure 2). This proves that through genetic engineering—in this case, transfecting BMSCs with BMP12—it is possible to stimulate their differentiation into tendon tissue cells [137].

In another study, researchers infected human TSPCs with a lentiviral vector to overexpress FGF2. They significantly enhanced tendon induction in vitro and improved tendon repair in vivo [90]. There was elevated expression of Tnmd, type I collagen and other tendon-associated proteoglycans in stem cells, confirming that Scx in BMSCs stimulates their differentiation into tenocytes [138]. An adenoviral vector was used to transfect human BMSCs with Mkx. Mkx has been demonstrated as a tendon transcription factor [48]. After transfection, these cells showed increased expression of the tendon-related genes type I collagen, Tnmd and Tnc [139]. The transcription factor Egr regulates collagen production and inhibits the expression of peroxisome proliferator-activated receptor gamma (PPARγ), Sox9 and Runx2 [140,141]. Ectopic expression of Egr1 in TSPCs by transfection of an Egr1-expressing plasmid induced tendon differentiation of these cells. Moreover, TSPCs overexpressing Egr1 promoted rotator cone repair in rabbits by activating the BMP12/Smad1/5/8 pathway [141]. 

Another promising direction that can be used to improve tendon regeneration is the use of non-coding RNAs such as microRNAs (miRNAs) and long non-coding RNAs (lncRNAs), which are involved in this process [36]. A miRNA inhibits the expression of a target gene by preventing translation of the transcript. The increase of miR-135a in TSPCs inhibited ROCK1 gene expression in senescent TSPCs, which inhibited senescence and enhanced tendinous differentiation [142]. In contrast to this study, the use of miR-378a knock-in transgenic mice showed that the absence of this molecule inhibited collagen and ECM production, tendon differentiation and tendon repair in vitro and in vivo [143]. A study showed that methylation of the promoter of the long non-coding RNA (lncRNA) Morf4l1 in damaged tendons led to a decrease in its expression and inhibition of tenocyte proliferation [144]. This molecule stimulated TGF-β2 via miR-145-5p. After co-incubating ASCs and injured tendon cells in vitro, Morf4l1 expression increased and the injured tenocytes began to divide. ASCs were injected into the injured tendon to repair the damaged tendon in vivo. Other lncRNAs can substantially influence cell differentiation and tissue regeneration [145,146]. Elevated levels of the lncRNA H19 in human TSPCs improved tendinous differentiation and promoted tendon repair in vivo in mice by activating the TGF-β1 signalling pathway [147]. Additional research is required to thoroughly understand the molecular mechanism underlying this process. Moreover, this direction promises the identification of new tendon-specific biomarkers. In summary, genetic modification has provided very promising results and it will likely support tendon regeneration in the future.

### 3.10. Biomaterials

#### 3.10.1. Effects of 3D Biomatrices on Cell Properties

In addition to growth factors, non-coding RNAs and signalling molecules, the microscopic environment—which comprises macromolecules with specific biomechanical, biophysical and biochemical properties—plays a considerable role in regulating cell migration, proliferation and differentiation. Various biomaterials, natural or synthetic, provide a 3D environment that promotes cell proliferation and ECM remodelling. Currently, the influence of the 3D environment is being extensively studied to determine its impact on the fate and molecular behaviour of stem cells, as previous studies have confirmed that this direction is promising [148]. The research showed that TSPCs maintained phenotypic abnormalities and experienced a significant decrease in their self-renewal capacity in a 2D culture environment, but preserved their differentiation potential. Three-dimensional structures also influence the expression of various genes in cultured cells [149]. When adult and foetal tenocytes were cultured in 3D, large distinctions in gene expression between these two developmental stages were found, with 542 genes being differentially expresse, whereas in a monolayer culture, adult and foetal tenocytes revealed only 10 significantly different genes. The properties of biomaterials (e.g., stiffness and pore size) regulate the function of cells placed in this environment and affect their differentiation. Researchers reported that a RADA nanofibrous hydrogel, which mimics the natural tendon matrix, slowed down the ageing process of human TSPCs, as manifested by enhanced expression of tenogenesis-related genes [148]. This finding demonstrates that the properties of the environment in which a tissue is located can significantly affect the differentiation and proliferation of the cells within it. Another study investigated the thermosensitive butane diisocyanate (BDI)-collagen hydrogel (BC hydrogel) as a promising alternative for cell carriers in cell-based regenerative therapies [150]. The BC hydrogel demonstrated support for the survival, proliferation and metabolic activity of TSPCs, along with satisfactory dimensional stability and biocompatibility. Visualisation revealed that TSPCs exhibited stretched morphologies with typical spindle cell shapes when cultured within the BC hydrogel. Additionally, the BC hydrogel facilitated the formation of capillary-like structures by human umbilical vein endothelial cells (HUVECs) within the hydrogel matrix. These findings collectively indicate that the thermosensitive BC hydrogel holds significant potential as an injectable carrier for delivering TSPCs in tendon tissue engineering applications. One of the natural 3D materials studied for its effect on tendon regeneration is decellularised tendons. This matrix prevented the production of bone markers such as Alp, Ocn and Runx 2, thus preventing differentiation into bone cells. In another study, a decellularised preparation from *Macaca mulatta* Achilles tendon contained Fmod and stromal cell-derived factor 1, which are characteristic of a tendon of natural origin [151]. Decellularised tendon matrix enhanced TGF-β3-centred human ASC tenogenesis and Scx expression [152].

#### 3.10.2. The Influence of Topographical Structure 

Another important factor that influences stem cell differentiation is the topographical structure. The mature natural tendon matrix presents a hierarchical structure with strongly parallel collagen fibres. Therefore, biomaterials with parallel topography are beneficial in inducing tendon differentiation [153]: they create microenvironments that mimic the topographical structure of natural tendons to facilitate stem cell differentiation towards the tendon lineage. Another research showed that tenogenesis utilising topographical structures could be enhanced by diminishing the expression of histone deacetylases (HDACs) via the inclusion of Trichostatin A (TSA), an HDAC inhibitor. TSCPs were cultured on aligned poly (l-lactic acid) (PLLA) fibres [154]. The results suggested that incorporating TSA into aligned PLLA fibres had an additional impact in directing tenogenic differentiation. This outcome was proved in a rat model, where the A-TSA scaffold enhanced the structural and mechanical properties of the regenerated Achilles tendon. The research illustrated that HDACs play a role in tenogenic differentiation on aligned fibre topography and combining HDAC inhibition with aligned topography could represent a more effective strategy for promoting stem cell tenogenesis. A subsequent study aimed to explore the impact of biomimetic COL1-CS (shell)/PLLA (core) fibres on the tenogenic differentiation of human MSCs in vitro. It was observed that the aligned COL1-CS/PLLA fibres prompted higher rates of cell spreading and proliferation compared to plain PLLA fibres. Additionally, there was a notable increase in the expression of tendon-associated genes such as Scx and Col1, along with elevated levels of tenomodulin protein. Animal studies conducted in a rat Achilles tendon repair model further confirmed the beneficial effects of COL1-CS/PLLA in facilitating the regeneration of tendon-like tissue. These findings suggest that COL1-CS/PLLA could serve as an effective scaffolding system for tendon repair applications [155,156]. A different research explored a magnetically responsive nanocomposite hydrogel comprising collagen type I (COL I) and aligned iron oxide nanoparticles (IOPs) for potential use in tendon tissue engineering [157]. The hydrogel, consisting of COL I combined with remotely aligned IOPs (A/IOPs) and human tendon-derived stem/progenitor cells (hTSPCs) (COL I-A/IOPs-hTSPCs), was prepared. The alignment of IOPs was induced under a remote magnetic field, resulting in the formation of a stable and anisotropic nanocomposite COL I-A/IOPs hydrogel upon gelation of COL I. Over time, evaluations were conducted on cell viability, proliferation, morphology, formation of cell rows and alignment of IOPs and hTSPCs. It was observed that the morphology of hTSPCs aligned with the orientation of the anisotropic COL I-A/IOPs hydrogel, exhibiting increased row formation compared to pristine COL I and COL-R/IOPs. Furthermore, a higher proliferation rate and significant upregulation of tendon gene markers were noted in hTSPCs cultured in the COL I-A/IOPs hydrogel compared to those in COL I-R/IOPs and COL I alone. This innovative approach directs stem cell behaviour without the need for exogenous growth factors or pre-aligned COL I fibres, suggesting that anisotropic nanocomposite hydrogels hold considerable promise for tendon tissue engineering applications. Studies have shown that the diameter of the fibrils is also important. Prostheses with nanofibres stimulated collagen and proteoglycan production and cell proliferation, while those with microfibres stimulated the expression of tendon markers, including type III and V collagen and Tnmd. The elongated cell phenotype is necessary for the tendon phenotype [158]. TSPCs controlled by a specially designed topographical surface resulted in elongated TSPCs induced by a parallel membrane with polydimethylsiloxane microarrays showing improved expression of tendon marker genes [159]. In addition, differentiation of elongated TSPCs towards the cartilaginous and adipogenic lineage on this topography was inhibited.

#### 3.10.3. Polycaprolactone (PCL) as a Matrix

PCL is a biodegradable polyester with a degradation rate of 2–4 years. It degrades into slightly acidic metabolites [160]. It offers the potential for the body to replace the scaffold with functional tissue without long-term residues. PCL is able to absorb greater forces and exhibits greater stiffness compared to other polymers such as polyglycolic acid (PGA), PLLA or poly(lactic-co-glycolic acid) (PLGA) [161,162]. The elasticity of PCL depends mainly on its molecular weight [163]. PCL is already used as a suture material or carrier for drug delivery systems [159]. In the literature, it has been studied as an implant material for tendons with variations in electrospinning or weave designs [164,165,166,167]; 3D-printed scaffolds based on PCL are a promising direction in the treatment of acute tendon injuries. In vitro experiments have shown that those scaffolds have properties that are desirable in tendon implantation materials. It appears that 3D-printed materials could be better than electrospun materials. Three-dimensional printing is more convenient than electrospinning when using PCL: biological and mechanical parameters can be better adjusted with 3D printing [168,169,170]. The functionality of 3D printing makes it a very promising method of obtaining matrices for reconstruction purposes.

A known problem in the use of PCL is its high hydrophobicity, which results in inhibition of cell growth on its surface [171]. Therefore, work is necessary to solve this problem. Currently, researchers are testing plasmochemical methods. These methods contribute to the formation of functional groups on the polymer surface, which reduces hydrophobicity and thus prevents growth inhibition on the PCL surface [172].

### 3.11. Mechanotransduction Stimulation

Mechanotransduction is a cascade of signalling pathways that guide cell differentiation and affect gene expression [173]. Research indicates that physical signals are key components of the in vivo niches of mesenchymal stem cells (MSCs) and play a significant role in determining MSC fate in various in vitro models. A tendon is a mechanosensitive tissue. It is constantly subjected to unilateral mechanical stretching; therefore, tendon cells retain their inherent morphology and tendon phenotype [174]. In addition, dynamic mechanical stimulation is required for full development of a tendon along with an advanced form of tendon matrix proteins (type I and III collagen) and stable expression of tendon-specific transcription factors such as Scx or tenascine C. The results suggested that cyclic stretching has influence on the process of tenogenic differentiation [175]. A study conducted on a porcine-derived acellular dermal matrix (PADM) demonstrated that applying tensile forces led to an increase in the expression of tenocyte markers. Specifically, scleraxis and tenascin C exhibited early increases that persisted throughout the duration of the study [176]. In a subsequent study, the effects of uniaxial and biaxial mechanical loading on TDSCs were investigated. Uniaxial loading prompted TDSCs towards both tenogenic and osteogenic differentiation, whereas biaxial loading led to osteogenic, adipogenic and chondrogenic differentiation. Additionally, the research revealed that uniaxial loading induced PKB (AKT) phosphorylation (pAKT), while biaxial loading induced pERK. Inhibition of the PI3K/AKT signalling pathway diminished tenogenic differentiation and tendon formation in 3D TDSC constructs subjected to uniaxial loading. Moreover, application of uniaxial loading on 3-dimensional (3D) TDSC constructs resulted in tenogenic-specific differentiation and neo-tendon formation, findings that were replicated in human TDSCs [104]. Other research [177] showed that the duration of mechanical stimulation affects the physical properties of callus and the migration of MSCs to the fracture site. Another study induced the differentiation of umbilical cord tissue MSCs towards tendon tissue solely by applying nanovibration (30–80 nm and a frequency of 1 kHz) and without the need for growth factors or complex scaffolds. Stem cells were grown on two-dimensional (2D) cell culture plates that were connected to piezoceramic systems. The results showed that nanovibration increased the expression of genes that encode tendon-related proteins, indicating that MSC mechanoregulation can be used in regenerative medicine [178].

Apart from the mechanical stimulation per se, the characteristic of the stimulation also hold significance. Different stimulation modes and parameters, including strain amplitude, loading frequency and intensity, have disparate effects on stem cell differentiation. Research shows that biaxial loading induces differentiation of osseous, adipogenic and cartilaginous tissue as was previously mentioned. Uniaxial cyclic tensile loading with an amplitude of 8% induced tendinous differentiation of BMSCs and protein and gene expression similar to primary tenocytes and inhibited differentiation in the non-tendinous lineage [179]. The intensity of loading is thought to affect cell differentiation. A moderate intensity tends to promote the expression of tendon-associated genes and the formation of neo-tendon, while a high intensity tends to lead to osseous differentiation [180]. In addition, different frequencies (0.3, 0.5 and 1.0 Hz) of cyclic stretching with the same amplitude have different effects on TPSC proliferation and expression of type I collagen, tenascin-C, Tnmd and Scx. The most pronounced tendon induction was obtained at a frequency of 0.5 Hz. The optimal unilateral loading parameters for tendinous differentiation of MSCs are a mechanical load with an amplitude of 2% and at a frequency of 0.1 Hz. It induces tendon differentiation of ASCs encapsulated in collagen hydrogel [181].

### 3.12. Pioglitazone and Its Effect on Stem Cells

In addition to the previously described factors that affect stem cells, drugs, including pioglitazone, have also shown promising effects. Pioglitazone exerts a hypoglycaemic effect by acting on PPARγ and is used to treat patients with diabetes. Moreover, this drug increases the proliferation of MSCs and the secretion of VEGF and collagen in these cells, as well as the expression of matrix genes and the secretion of collagen by tenocytes [182]. The use of drugs to generate molecules that affect the pathways activated during the tendon regeneration process has prospects for clinical application. Table 2 summarises the properties and potentially important functions of the factors previously mentioned in this article.

## 4. Conclusions

Recent research indicates that the use of stem cells, including activation of the relevant pathways that stimulate their differentiation, is a future direction to accelerate tendon regeneration. This approach produces tendon tissue with an improved blood supply and enhanced histological and biomechanical properties. This process could be influenced by gene and cell therapy, PRP, growth factors, tissue engineering and 3D printing to allow patients to recover more quickly from tendon injuries and to reduce the risk of re-injury. However, to fully develop specific treatment regimens that can be applied on a large scale, additional research in this direction is needed to predict the effects of such novel therapies.

## Figures and Tables

**Table 1:** The advantages and disadvantages of stem cells used in tissue engineering.

Type of Stem Cells	Advantages	Disadvantages	Refs.
Tendon progenitor stem cells (TSPCs)	Differentiation into tissue similar in structure to tendons	Need to obtain a large volume of source material to obtain an adequate number of cells	[24,25,26,27]
Adipose tissue-derived stem cells (ASCs)	Easy to obtain through liposuction	Tendency to undergo adipogenesis Fat deposition during the process of tendon regeneration	[28,29,30,31,32]
Bone marrow-derived mesenchymal stem cells (BMSCs)	Easy to obtain	Occurrence of ossification in tendons	[33,34,35,36,37,38,39]
Embryonic stem cells (ESCs)	Ability to differentiate into all cell lines	Ethical concerns due to the way they are obtained Risk of neoplastic transformation	[40]
Induced pluripotent stem cells (iPSCs)	Ability to differentiate into all cell lines	Risk of neoplastic transformation	[42,43]

**Table 2:** Methods used in tissue engineering for tendon reconstruction.

Category	Factors	Effect on the Cell/Tissue and Mode of Action	Refs.
Growth factors	Transforming growth factor beta (TGF-β)	Participates in inflammatory reactions, angiogenesis, collagen synthesis and fibrosis or excessive scarring Activates the Smad2/3 intracellular pathway	[51,52,53]
	Connective tissue growth factor (CTGF)	Activates focal adhesion kinase (FAK)/extracellular signal-regulated kinase 1/2 (ERK1/2) pathway, promotes proliferation of damaged cells and stimulaties expression of tendon markers such as Scx and Tnmd	[28,81,82,83]
	Fibroblast growth factors (FGFs)	Activates the FGF/ERK/mitogen-activated protein kinase (MAPK) pathway affectes cell migration, proliferation and differentiation	[88]
	Growth differentiation factors (GDFs)	Participates in the development of the musculoskeletal system inhibits fibrosis	[73,74,80]
	Insulin-like growth factor 1 (IGF-1)	Activates the phosphoinositide 3-kinase (PI3K)/Akt, Ras-MAPK and phospholipase C (PLC) kinase pathways	[103,104,105]
Proteins	Yes-associated protein (YAP)	Increases the production tendon-related proteins such as tenomodulin and tenascin C	[132]
Wnt ligands	Wnt ligands	Mediate tendon differentiation of mesenchymal stem cells induced by mechanical stimulation	[47,135]
Genetic modification	microRNA-135a (miR-135a)	Inhibits ROCK1 gene expression and stimulates tendon differentiation	[142]
RNA	Long non-coding RNA (lncRNA) H19	Activates TGF-β1 signalling pathway Stimulates tendon differentiation and tendon repair.	[147]
3D biomatrix	3D biomatrix	Promotes cell proliferation and extracellular matrix remodelling	[148]
Drugs	Pioglitazone	Simulates peroxisome proliferator-activated receptor gamma (PPARγ), vascular endothelial growth factor (VEGF) and collagen secretion	[182]

## Data Availability

Not applicable.
